# Tunable Electrical Properties of Cobalt-Doped Maghemite Nanoparticles for Advanced Resistive and Thermistor Applications

**DOI:** 10.3390/nano15070534

**Published:** 2025-04-01

**Authors:** Mokhtar Hjiri, Sonia Soltani, Anouar Jbeli, Nazir Mustapha, Nouf Ahmed Althumairi, Majdi Benamara, Manuel Almeida Valente

**Affiliations:** 1 Department of Physics, College of Sciences, Imam Mohammad Ibn Saud Islamic University (IMSIU), Riyadh 11623, Saudi Arabia; mbhjiri@imamu.edu.sa (M.H.); nmmustapha@imamu.edu.sa (N.M.); 2 Department of Physics, College of Science, Qassim University, Buraidah 51452, Saudi Arabia; s.soltani@qu.edu.sa; 3 Department of Physics, College of Science, Majmaah University, Al-Majmaah 11952, Saudi Arabia; a.jbeli@mu.edu.sa (A.J.); n.althumairi@mu.edu.sa (N.A.A.); 4 i3N and Physics Department, University of Aveiro, Campus Universitário de Santiago, 3810-193 Aveiro, Portugal; mav@ua.pt

**Keywords:** cobalt-doped maghemite, charge transport dynamics, impedance spectroscopy, electrical conductivity, nanostructured ceramics

## Abstract

Maghemite (γ-Fe_2_O_3_) nanoparticles have attracted considerable interest for electronic applications due to their tunable electrical properties. Doping strategies offer an effective way to optimize their resistive behavior for use in electronic devices. In this study, cobalt (Co) was incorporated into γ-Fe_2_O_3_ to enhance its resistive properties. X-ray diffraction (XRD) confirmed the retention of the cubic P4_3_32 phase, with Co doping inducing subtle lattice distortions due to ionic substitution. Scanning and transmission electron microscopy (SEM/TEM) revealed morphological changes, where Co incorporation influenced particle shape and size distribution. Electrical conductivity analysis demonstrated a decrease in both AC and DC conductivity with the increase in Co content, indicating enhanced resistive behavior. The increase in activation energy suggests a reduction in charge carrier mobility, leading to higher resistivity. Impedance spectroscopy further confirmed increased real and imaginary impedance values, reinforcing the role of Co in suppressing charge transport. These results position cobalt-doped maghemite as a promising material for electronic resistive devices, such as tunable resistors and negative temperature coefficient (NTC) thermistors, where controlled conductivity and stable resistive behavior are essential.

## 1. Introduction

Materials exist in various structural forms, including nanoparticles, ceramics, and nanostructures, each exhibiting distinct properties that make them suitable for a wide range of technological applications [1,2,3,4,5]. Nanoparticles have gained significant attention due to their high surface area-to-volume ratio and size-dependent quantum effects, enabling enhanced reactivity, tunable optical properties, and improved mechanical strength. These features make them indispensable in fields such as biomedicine, catalysis, energy storage, and environmental remediation. Metal oxide nanoparticles, such as ZnO, Fe_2_O_3_, and TiO_2_, are widely used in photocatalysis, antimicrobial coatings, and electronic devices due to their stability and semiconducting properties, while functionalized nanoparticles play a crucial role in targeted drug delivery and biosensing applications [6,7,8,9]. Ceramics, known for their exceptional mechanical strength, thermal stability, and electrical insulation properties, are essential to high-temperature applications, structural components, and energy conversion systems. Advanced ceramics, such as perovskite oxides, alumina, and zirconia, are extensively employed in fuel cells, superconductors, and aerospace industries due to their superior hardness and corrosion resistance. Additionally, ceramic-based dielectric materials are widely utilized in capacitors, sensors, and high-frequency electronic devices for their excellent insulation properties and tunable permittivity [10,11]. Recent research has focused on modifying ceramic properties through doping and nanostructuring to enhance their ionic conductivity and electrochemical performance, broadening their applicability in energy storage and electronic applications [12,13,14,15]. Meanwhile, nanostructures, including nanowires, nanotubes, and thin films, have revolutionized materials science by offering remarkable electronic, optical, and mechanical properties that differ significantly from their bulk counterparts. Semiconductor nanowires are being explored for next-generation transistors, solar cells, and quantum computing due to their superior charge carrier mobility and tunable bandgap, while carbon nanotubes and graphene-based materials are being integrated into flexible electronics, high-performance batteries, and electromagnetic shielding applications [16,17]. The development of nanostructures has also paved the way for highly efficient sensors, photonic devices, and miniaturized circuits, driving the advancement of modern technology. By leveraging the unique advantages of these material forms, researchers are continuously developing innovative strategies to optimize their properties and expand their functionality for cutting-edge applications, with a strong focus on synthesis methods, structural modifications, and dopant engineering to tailor materials for specific industrial and scientific needs [18,19,20]. The regulation of electron properties in nanomaterials is key to controlling charge transport, band structure, and conductivity [21,22]. Doping and structural modifications influence carrier mobility and electronic states, while external factors like temperature and electric fields provide further tunability for electronic applications [23,24].

Nanoparticles have played a pivotal role in advancing nanotechnology due to their unique size-dependent properties [25,26]. Among these, metal oxide nanoparticles stand out for their exceptional attributes, such as high surface area-to-volume ratio, super-paramagnetism, ease of separation, and enhanced surface properties [27,28]. These characteristics make them highly promising for a wide range of applications, including lithium-ion batteries, ferrofluids, microwaves, photocatalysts, magnetic data storage, gas sensors, spintronics, and noise filters [29]. Metal oxides are particularly notable for their chemical and thermal stability, derived from the formation of ionic bonds between positive metal ions and negative oxygen ions. Their partially filled d-shells also confer special electronic properties useful in various devices [30]. These properties include reactive electronic transitions, high dielectric constants, bandgaps, and qualities beneficial for photonic, electrochromic, and superconducting applications. Despite these advantages, achieving precise control over the crystallization of metal oxide nanoparticles to obtain specific physiochemical features remains challenging [31,32,33,34,35].

Iron oxide nanoparticles, especially those composed of magnetite (Fe_3_O_4_) and maghemite (γ-Fe_2_O_3_), have garnered significant interest due to their crystalline structures, diverse oxidation states, magnetic properties, cost-effectiveness, and environmental friendliness [36,37]. Various synthesis methods (such as co-precipitation [38]; hydrothermal [39], solvothermal [40], and thermal decomposition [41]; and the sol–gel process [42]) have been employed to produce these nanoparticles for applications in drug delivery, magnetic data storage, electronic devices, and biodetection [43,44]. The sol–gel process is particularly advantageous for producing metal oxide nanoparticles, including iron oxide, due to its ability to create uniformly sized and characterized nanoparticles with high homogeneity [14,45]. This method is more cost-effective than others, facilitating large-scale production while minimizing contaminants, resulting in high-purity nanopowders. Overall, the sol–gel process provides a reliable and efficient means of producing metal oxide nanoparticles with precise control, low cost, and high purity, which is beneficial for various applications in materials science, electronics, biology, and energy storage [46,47].

Research has shown that incorporating appropriate dopants, such as other metal oxides and noble metals, can enhance the physical properties of nanoparticles. Recent studies have demonstrated that doping with rare earth metals, such as samarium, europium, magnesium, and cobalt ions, can control nanoparticle size and improve magnetic, electrical, and optical properties [48,49]. However, the potential of magnesium doping in maghemite nanopowders for dielectric field applications remains largely unexplored. This study aims to fabricate maghemite (γ-Fe_2_O_3_) and cobalt-doped maghemite nanoparticles by using the sol–gel process. The microstructural properties were analyzed by using X-ray diffraction (XRD), and morphological analysis was conducted through scanning and transmission electron microscopy (SEM/TEM). Electrical characterization studies were performed over a temperature range.

## 2. Materials and Methods

### 2.1. Synthesis of Pure and Cobalt-Doped Maghemite (γ-Fe_2_O_3_) Nanopowders

To prepare maghemite (γ-Fe_2_O_3_) samples, we began by dissolving a specific amount of iron precursor in a predetermined volume of methanol. This mixture was stirred for a defined period to ensure proper dissolution, after which we added a larger volume of ethyl alcohol to facilitate drying. The resulting solution was transferred to an autoclave, where it underwent a heating process under supercritical conditions of ethyl alcohol, characterized by its critical temperature (T_c_) and pressure (P_c_).

For synthesizing cobalt-doped γ-Fe_2_O_3_ samples, we replicated the initial steps, incorporating the cobalt precursor (C_6_H_9_CoO_6_·2H_2_O) at varying concentrations during the stirring process. This allowed us to investigate the impact of different doping levels on the resulting material properties. The pure and cobalt-doped iron oxide nanoparticles were then subjected to a heat treatment at a specific temperature for a set duration in an air environment. Each sample was designated with a specific naming convention reflecting the nominal cobalt content (Co0FO, Co1FO, Co3FO, and Co5FO), which facilitated the study of its structural and magnetic characteristics, including changes in superparamagnetic behavior, crystallinity, and particle size distribution as a function of cobalt doping. These characteristics were analyzed to understand how cobalt influences the magnetic and electrical properties of maghemite, particularly regarding superparamagnetic behaviors (SCD), which are crucial to various applications.

### 2.2. Characterization

To characterize the nanoparticles, we utilized various instruments and techniques. Microstructural properties were analyzed by using X-ray diffraction (XRD) with a Bruker D8 Advance A 25 diffractometer (Bruker, Billerica, MA, USA), employing a CuKα radiation source (*λ* = 1.5406 Å) at 40 kV. Morphological properties were assessed via scanning electron microscopy (SEM) by using a ZEISS GeminiSEM 460 (Carl Zeiss AG, Oberkochen, Germany). Additionally, transmission electron microscopy (TEM) with a JEM 2200 FS (JEOL Ltd., Tokyo, Japan) at 200 kV was used to further evaluate the shape, size, and structure of the nanoparticles. Energy-dispersive X-ray (EDX) characterization detectors integrated with both SEM and TEM were employed to determine the chemical composition of the samples. For dielectric measurements, a comprehensive system was used, which included a microcomputer, a cryostat, a vacuum system for enhanced thermal insulation, and various measuring devices.

Impedance spectroscopy was conducted by using a specialized analyzer, with measurements taken across a defined temperature range and frequency spectrum. The preparation of the samples involved pressing nanoparticle powders into standardized pellets, which were then fitted with electrodes to facilitate electrical measurements. All electrical experiments were carefully monitored and controlled through dedicated software, with temperature regulation implemented to ensure consistent experimental conditions. Additionally, the measurements were performed in a controlled environment to improve thermal conductivity and minimize humidity-related issues.

## 3. Results and Discussion

### 3.1. Structural Behaviors

**Figure 1:**
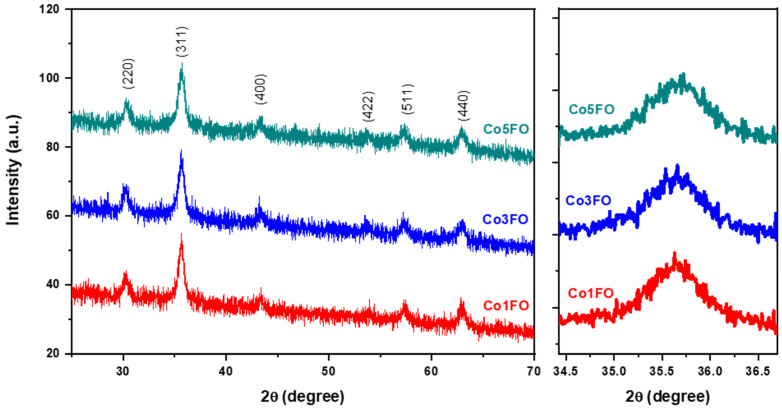
XRD patterns of cobalt-doped maghemite nanoparticles with a zoomed-in view of the indexed peak (311) on the right side.

The microstructure of the annealed nanopowders was investigated by using X-ray diffraction (XRD). The reflection peaks observed in Figure 1 for the (220), (311), (400), (422), (511), and (440) planes correspond to maghemite (γ-Fe_2_O_3_), as identified by JCPDS card number 39-1346 [50]. The pure and cobalt-doped maghemite samples exhibited excellent purity and no discernible secondary phases, with a P4_3_32 space group and a cubic crystalline structure [51]. Additionally, cobalt was uniformly distributed throughout the lattice without forming discrete phases. To assess the impact of cobalt doping on crystallite size, the average crystallite size (*D*) was determined by using the Scherrer formula:

(1) D=0.9 λβcos⁡θ.

By using an incident wavelength (*λ*) of 1.5406 Å, we measured the X-ray peak width (*β*) on the 2*θ*-axis, known as the full width at half maximum (FWHM). The Bragg angle (*θ*), expressed in degrees, was also considered. We fit the (311) peak of each sample by using a Gaussian equation to determine the Bragg angle (*θ*) and FWHM values. This analysis supports the conclusion that cobalt doping influences particle size. The average crystallite size for the doped samples ranged between 11 and 14 nm. The increase in crystallite size can be attributed to the incorporation of cobalt into the lattice, as cobalt has a larger ionic radius (r (Co^3+^) = 74.5 pm) [52] compared with iron (r (Fe^3+^) = 67 pm) [53]. The lattice parameter of the cubic structure and the cell volume can be determined from the indicated peaks by using the following equations:

(2) a= λ h2+k2+l22sin⁡θhkl

(3) $$V=a^{3}$$

where (*hkl*) are the Miller indices. The lattice parameters of the doped maghemite nanoparticles ranged between 8.330 and 8.372 Å, while the unit cell volume varied from 578.1 to 587.8 Å^3^.

Cobalt doping in maghemite (γ-Fe_2_O_3_) induces significant structural changes that directly impact its electrical properties. The expansion of the lattice parameter (ranging from 8.330 to 8.372 Å) and the increase in unit cell volume (from 578.1 to 587.8 Å^3^) confirm the successful incorporation of Co^3+^ ions into the maghemite structure. This incorporation leads to a distortion in the crystal lattice due to the larger ionic radius of Co^3+^ (74.5 pm) compared with Fe^3+^ (67 pm). Additionally, the increase in crystallite size (up to 16 nm) reduces grain boundary density, which typically facilitates charge transport. However, cobalt doping disrupts the natural hopping conduction mechanism between Fe^2+^ and Fe^3+^ ions, leading to a reduction in electrical conductivity and an increase in resistance. The presence of Co^3+^ alters the charge transfer dynamics by introducing localized electronic states that act as trapping centers, hindering carrier mobility. Furthermore, cobalt substitution can decrease the number of available Fe^2+^/Fe^3+^ pairs responsible for polaronic conduction, further impeding electron transport. As a result, the overall electrical resistance of maghemite increases with cobalt doping, making it less conductive compared with the undoped material. Furthermore, a noticeable shift in the XRD peak positions, particularly for the indexed (311) peak, is observed in Figure 1. This shift can be attributed to the structural strain introduced by cobalt doping, which leads to modifications in the unit cell dimensions. The strain arises due to the size mismatch between Co^3+^ and Fe^3+^ ions, resulting in lattice distortions that affect the diffraction pattern [54].

### 3.2. Morphological and Chemical Composition Properties

**Figure 2:**
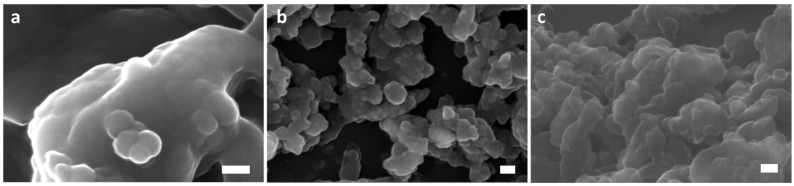
SEM images of (**a**) Co1FO, (**b**) Co3FO, and (**c**) Co5FO with a scale of 100 nm.

Scanning electron microscopy (SEM) was employed to investigate the morphological properties of cobalt-doped maghemite (γ-Fe_2_O_3_) nanopowders. Figure 2a, Figure 2b and Figure 2c present SEM micrographs corresponding to the Co1FO, Co3FO, and Co5FO samples, respectively, where the numbers indicate the increasing cobalt doping concentration.

From the images, the nanopowders exhibit fine grains with a high degree of porosity. The particles appear to have a spherical or quasi-spherical morphology, with a tendency to form agglomerates due to interparticle interactions. As the cobalt doping concentration increases, a noticeable reduction in grain size is observed, likely due to the influence of cobalt ions on the nucleation and growth process during synthesis.

In Figure 2a (Co1FO), the sample shows relatively large agglomerates, with well-defined primary particles appearing on the surface. The morphology suggests a more heterogeneous distribution, possibly due to lower cobalt content, which does not significantly alter the grain growth kinetics.

In Figure 2b (Co3FO), the particles are more uniformly distributed with slightly reduced sizes compared with Co1FO. The surface texture appears rougher, indicating a transition towards finer nanoparticles with enhanced porosity. In Figure 2c (Co5FO), the sample exhibits the smallest grain size among the three compositions, with increased agglomeration. The fine particle distribution and porosity suggest that cobalt doping influences the crystallization dynamics, reducing the effective particle size.

**Figure 3:**
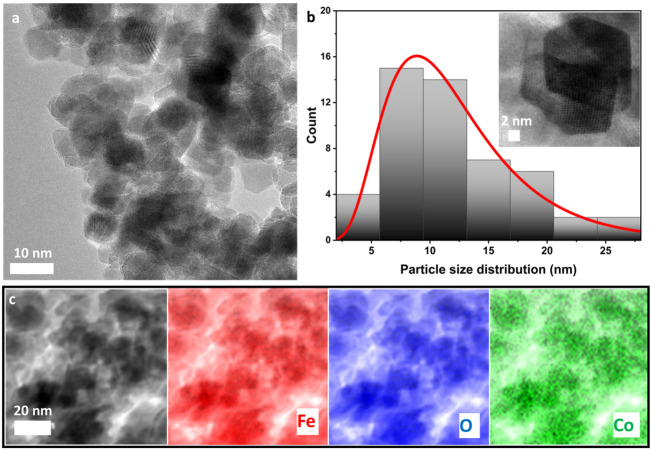
TEM image of Co3FO nanoparticles at the scale of (**a**) 10 nm and (**b**) a particle size distribution diagram with a TEM image of a hexagonal-shaped particle at a scale of 2 nm (inset). (**c**) The elemental mapping of the Co3FO sample.

To further investigate the shape and size of cobalt-doped maghemite nanoparticles, transmission electron microscopy (TEM) was utilized. Figure 3a displays a low-magnification TEM image of the Co3FO sample, revealing an agglomeration of ultrafine nanoparticles. The particles exhibit a range of morphologies, including spherical, hexagonal, and cubic shapes, indicating the coexistence of different crystallographic orientations. The agglomeration may be attributed to magnetic dipole interactions among the particles, as well as van der Waals forces, which are common in magnetic nanoparticles. The observed size variation suggests that the synthesis conditions allow for the formation of multiple crystalline facets, contributing to the shape diversity. To determine the average particle size of the Co3FO nanoparticles, ImageJ software (version 1.53) was used for particle size analysis, as depicted in Figure 3b. The histogram illustrates the particle size distribution, which follows a Gaussian-like curve, with the average particle size calculated as 9 nm. The relatively narrow size distribution confirms that the synthesis method employed produces nanoparticles with controlled growth. The nanoscale size of these particles is particularly advantageous for applications in nanomagnetism, catalysis, and electronic materials, where surface effects and quantum confinement play a significant role.

To gain further insights into the structural characteristics, high-resolution TEM (HRTEM) images were obtained, as shown in the inset of Figure 3b. The image distinctly highlights a hexagonal nanoparticle, where well-defined lattice fringes are clearly visible, confirming the crystalline nature of the sample. These fringes correspond to the atomic planes of maghemite (γ-Fe_2_O_3_), verifying the high structural order at the nanoscale. The presence of hexagonal shapes suggests that cobalt doping may influence the crystal growth process, leading to preferential formation along specific crystallographic orientations. This structural modification could impact the magnetic and electrical properties of the nanoparticles, making them suitable for advanced applications in electronic devices and energy storage.

To confirm the elemental composition and spatial distribution of elements in the sample, energy-dispersive X-ray spectroscopy (EDX) mapping was performed. The results are shown in Figure 3c, where Fe (red), O (blue), and Co (green) are mapped. The homogeneous distribution of Fe and O throughout the sample indicates that the fundamental structure of maghemite remains intact after doping. Additionally, the Co mapping demonstrates that cobalt is successfully incorporated into the maghemite lattice, with no visible phase segregation, suggesting the effective substitution of Fe by Co atoms. This uniform distribution is crucial to ensuring consistent modifications in the electrical and magnetic properties across the entire nanoparticle ensemble.

**Figure 4:**
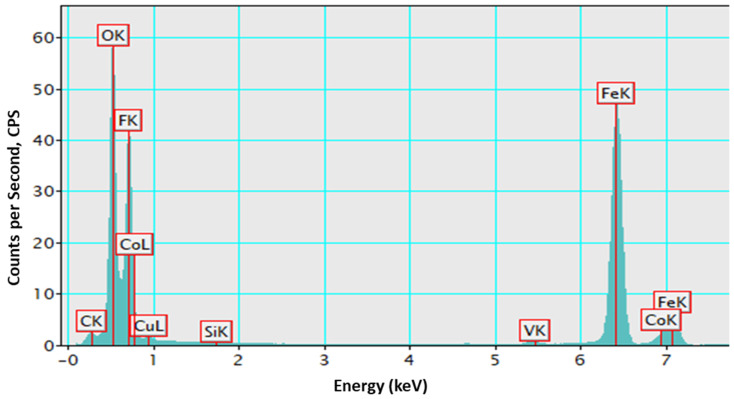
EDX spectrum of the Co3FO sample.

The electronic structural properties of materials are closely linked to their elemental composition and distribution, which play a crucial role in defining their physical and chemical behavior [55]. Energy-dispersive X-ray spectroscopy (EDX) is a powerful technique for identifying the elemental makeup of a material, providing insights into its structural integrity and doping efficiency. To further validate the chemical composition of the Co3FO sample, an EDX spectrum was recorded, as shown in Figure 4. The spectrum exhibits strong peaks corresponding to Fe and O, confirming the maghemite phase. A distinct peak for Co is also observed, verifying the successful incorporation of the dopant element. The relatively low intensity of the Co peak is expected due to the 3% doping level, indicating its presence in minor yet detectable quantities. Additionally, small peaks corresponding to C and Si are detected, which are likely attributed to the TEM grid or residual impurities from the synthesis process. The presence of F may result from precursor remnants or surface contamination. These results confirm the elemental composition of the sample and support the successful doping of cobalt into the maghemite structure.

### 3.3. Electric Behaviors

#### 3.3.1. AC Conductivity

**Figure 5:**
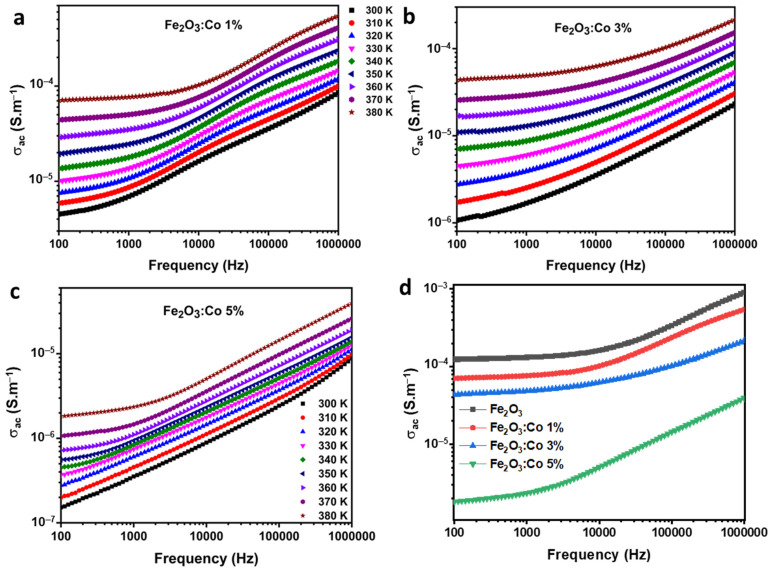
Conductivity spectra of (**a**) Co1ZO, (**b**) Co3ZO, and (**c**) Co5ZO over a range of temperatures. (**d**) Conductivity curves of pure and Co-doped Fe_2_O_3_ at 380 K.

Figure 5 depicts the variation in conductivity (*σ*) of the prepared samples as a function of frequency across the temperature range of 300–380 K. The conductivity was determined by using the following formula:

(4) σ=GeS ,

where *S* is the surface area, *G* is the conductance, and e is the thickness of the materials. The doped samples exhibited two distinct conductivity regions: AC and DC. In the low-frequency range of 100 Hz to 1000 Hz, the DC conductivity region remained almost constant, attributed to a reduction in ion jumping motion due to grain boundaries impeding charge carrier mobility [56]. This indicates slower charge carrier mobility between Fe^3+^ and Fe^2+^ ions at low frequencies [57]. Conversely, for frequencies above 10 kHz, a sharp exponential increase in AC conductivity (*σ_ac_*) was observed. This behavior is linked to dispersion phenomena, particularly relaxation and reorientation processes described in hopping relaxation models. At high frequencies, the conductivity of ferrite materials can be explained by two mechanisms: the large polaron jump and the small polaron hop (SPH) [58].

The SPH mechanism, which increases *σ_ac_* with frequency, was evident in our samples. All samples also showed increased conductivity with temperature, confirming their semiconducting nature. The overall conductivity (*σ*) can be described by Jonscher’s universal power law:

(5) $$\sigma \left(\omega \right)=\sigma_{dc}+A\omega^{n}\;,$$

where *σ_dc_* and *σ_ac_* are the conductivity components at low and high frequencies, A is a constant indicating the degree of interaction among mobile entities, and n is a power exponent between 0 and 1. Increasing cobalt (Co) content was found to decrease conductivity due to impurity-induced disruptions in atomic arrangement, increased electron scattering, charge localization, and alterations in the electronic band structure [59]. Similar findings have been reported in studies on Co-doped ferrites.

Figure 5d presents a comparative analysis of the AC conductivity of pure and Co-doped maghemite samples at various concentrations (1%, 3%, and 5%) over a wide frequency range. As shown, the undoped Fe_2_O_3_ sample exhibits the highest conductivity, while increasing Co doping systematically reduces conductivity across all frequencies. This trend is attributed to the structural distortions and defect-induced electron scattering caused by Co incorporation, which disrupts charge transport pathways. At low frequencies, all samples exhibit nearly constant conductivity corresponding to the DC region, suggesting limited charge carrier mobility due to grain boundary effects. In contrast, the high-frequency region shows a strong frequency-dependent increase in conductivity, consistent with the small polaron hopping (SPH) model. Notably, the Co 5% sample demonstrates the lowest conductivity, indicating a pronounced effect of doping on charge localization and band structure modifications. These findings align with previous reports on transition metal-doped ferrites, where dopant-induced lattice distortions lead to reduced charge carrier mobility.

#### 3.3.2. Activation Energy

**Figure 6:**
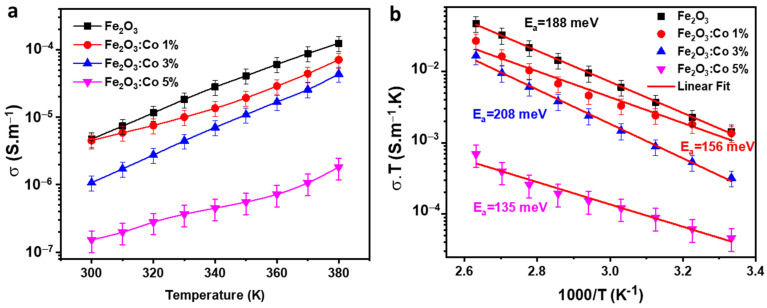
(**a**) Temperature dependence of *σ_dc_* and (**b**) *σ_dc_*·*T* vs. 1000/T plots for the CoFO samples.

Figure 6a illustrates the variation in *σ_dc_* with temperature for all compounds. As the temperature increases, *σ_dc_* also rises, indicating that our compounds exhibit semiconducting behavior [60]. The lower conductivity of Fe_2_O_3_:Co 5% compared with Fe_2_O_3_:Co 3% is likely due to increased charge carrier localization and scattering effects caused by excessive Co doping. At 3% doping, Co ions enhance charge transport by modifying the Fe^3+^/Fe^2+^ ratio, but at 5%, they introduce more defect states and structural distortions, which hinder polaron hopping. Additionally, high doping levels may reduce free carrier density and potentially lead to phase segregation, further disrupting conduction pathways and lowering overall conductivity. This temperature-dependent change in *σ_dc_* is attributed to the thermal activation of charge carriers within our samples and can be described by the Arrhenius equation [61,62]:

(6) σdc×T=σ0×exp−EakB×T 

In this equation, *E_a_* represents the activation energy, *σ*_0_ is the pre-exponential factor, k_B_ is Boltzmann’s constant, and *T* is the absolute temperature. In ferrite materials, electrical conduction typically occurs through the hopping mechanism of charge carriers between two different sites. The activation energy (*E_a_*) can be determined from the slope of the Log(*σ*·*T*) versus 1000/T plots shown in Figure 6b.

The calculated *E_a_* values for the lower- and higher-temperature ranges are 188 meV, 156 meV, 208 meV, and 135 meV for the doping levels of 0%, 1%, 3%, and 5%, respectively. This indicates that conduction in our samples is due to the hopping mechanism between Fe^3+^ and Fe^2+^ ions. The obtained *E_a_* values imply a significant potential barrier (W), which can hinder the mobility of charge carriers between sites. Given all the obtained *E_a_* values, the predominant conduction mechanism is thought to be small polaron hopping [63].

The variation in *E_a_* with the increase in Co doping highlights key modifications in charge transport properties. The decrease in *E_a_* for the 1% Co-doped sample (156 meV) compared with the undoped Fe_2_O_3_ (188 meV) suggests that at low doping levels, cobalt ions may facilitate charge carrier transport by altering the electronic structure and reducing localized trapping sites, possibly due to slight modifications in the Fe^3+^/Fe^2+^ ratio or an increase in lattice disorder, which enhances polaron mobility. However, at a moderate doping concentration of 3%, *E_a_* increases to 208 meV, indicating that the introduction of Co ions disrupts long-range charge transport. This effect is likely caused by increased structural distortions and additional defect states that act as scattering centers, increasing the energy required for small polaron hopping and leading to a more localized electronic environment that hinders carrier mobility. For the highest doping level (5% Co), *E_a_* decreases again to 135 meV, suggesting a competing effect where Co significantly alters the Fe_2_O_3_ lattice, possibly inducing new conduction pathways through percolation-like behavior, where dopant-induced defects facilitate localized hopping conduction. Additionally, the observed reduction in *E_a_* at this level might be associated with a shift in the density of states near the conduction band, leading to modified hopping distance and carrier interaction energy. These observations confirm that Co doping has a non-monotonic effect on charge transport, initially enhancing carrier mobility at low doping, increasing charge localization at moderate doping, and potentially reintroducing alternative conduction pathways at higher doping levels. These behaviors align with previous findings on transition metal-doped ferrites, where dopants influence the polaron binding energy and the strength of electron–phonon interactions, ultimately shaping the conduction mechanism in these materials.

#### 3.3.3. Electrical Impedance Studies

##### Real Part of Impedance (Z′)

**Figure 7:**
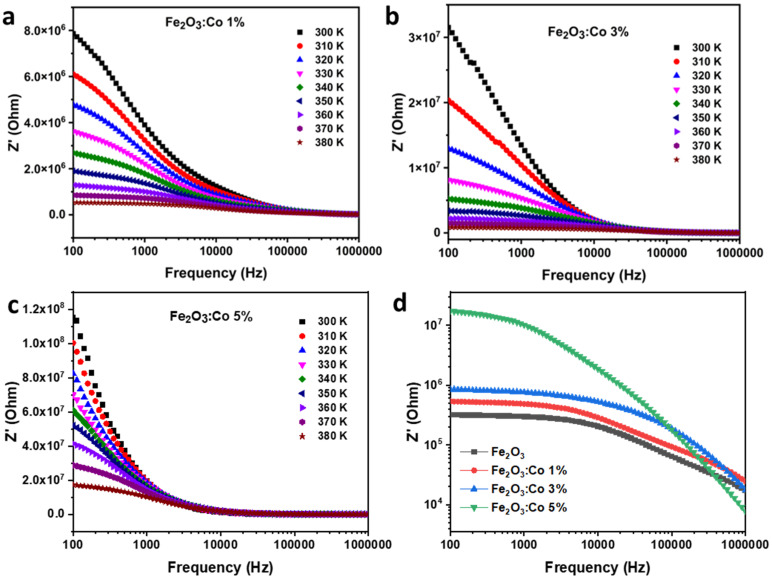
Frequency dependence of Z′ for (**a**) Co1ZO, (**b**) Co3ZO, and (**c**) Co5ZO. (**d**) Comparison between pure Co-doped samples at 380 K.

The impedance behavior of Co-doped γ-Fe_2_O_3_ is strongly influenced by both doping concentration and temperature. Figure 7a shows the real impedance (Z′) of the 1% Co-doped sample as a function of frequency at different temperatures (300–380 K).

At low frequencies, Z′ decreases gradually, while at high frequencies, it drops sharply due to space charge polarization. By comparing Figure 7a and Figure 7b, which represent 1% and 3% Co-doped samples, respectively, it is evident that Co doping increases impedance. This suggests that Co introduces localized defect states acting as charge carrier traps, reducing charge mobility and increasing resistivity. Additionally, impedance decreases with the increase in temperature in both Figure 7a and Figure 7b, indicating thermally activated conduction, where thermal energy facilitates charge carrier movement, overcoming the trapping effects caused by Co doping. A detailed comparison between Figure 7a and Figure 7b shows that increasing the Co content from 1% to 3% significantly increases Z′, likely due to enhanced lattice distortions and defect formation. The higher impedance values of the 3% Co-doped sample suggest greater suppression of charge transport, as excessive doping introduces more scattering centers, which hinder electron mobility. Figure 7c, which also represents the 3% Co-doped sample, confirms this trend by showing a consistent reduction in impedance with the increase in temperature. Overall, Figure 7a, Figure 7b and Figure 7c demonstrate that Co doping effectively tunes the electrical properties of γ-Fe_2_O_3_, with higher doping levels leading to a more resistive material, making it suitable for applications requiring controlled impedance behavior [64].

Figure 7d presents a comparative analysis of the real impedance (Z′) for pure and Co-doped γ-Fe_2_O_3_ samples with varying doping concentrations (1%, 3%, and 5%) as a function of frequency. The observed trend indicates that Co doping leads to a significant increase in impedance, particularly at lower frequencies, reinforcing the notion that cobalt incorporation induces defect states that act as charge carrier traps, thereby reducing charge mobility. The pure γ-Fe_2_O_3_ sample exhibits the lowest impedance, highlighting its higher electrical conductivity compared with the doped samples. Among the Co-doped samples, the 5% Co-doped γ-Fe_2_O_3_ exhibits the highest impedance, suggesting that excessive doping exacerbates charge carrier scattering due to increased lattice distortions and defect-induced barriers. Furthermore, the impedance behavior at high frequencies shows a convergence trend across all samples, implying that at these frequencies, the contribution of grain boundary resistance diminishes and bulk conduction becomes dominant. This comparative analysis underscores the tunability of γ-Fe_2_O_3_’s electrical properties through Co doping, making it a promising candidate for applications requiring controlled resistivity and impedance behavior.

##### Imaginary Part of Impedance (Z″)

**Figure 8:**
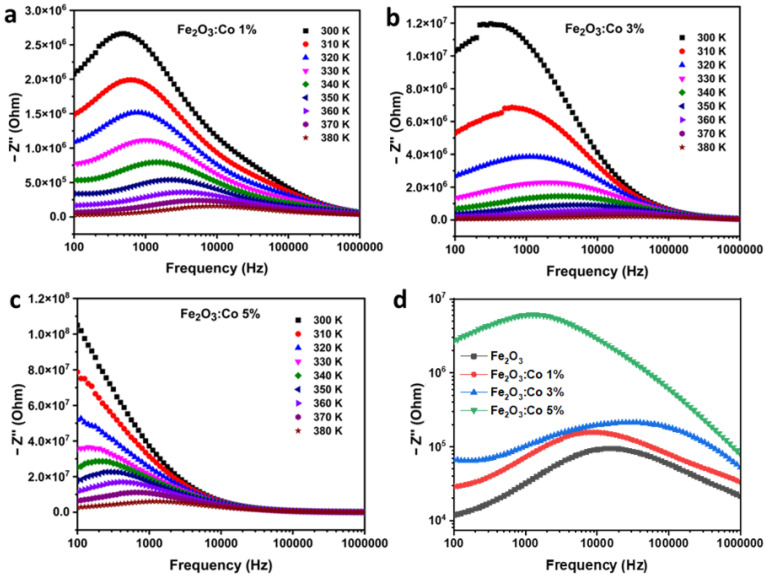
Frequency dependence of the imaginary part of the electrical impedance for (**a**) Co1ZO, (**b**) Co3ZO, (**c**) Co5ZO and (**d**) a comparison at 380 K between pure and Co-doped samples.

The imaginary part of impedance (Z″) for Co-doped γ-Fe_2_O_3_ at different doping concentrations (1%, 3%, and 5%) is presented in Figure 8a–c. In all cases, Z″ initially increases at low frequencies, reaches a peak, and then decreases with further frequency increases. This peak corresponds to the characteristic relaxation process within the material, where charge carriers respond to the applied AC field. As observed in Figure 8a and Figure 8b, increasing Co doping from 1% to 3% results in a broader and less distinct peak, indicating a more complex relaxation process due to increased defect sites and charge carrier scattering.

The temperature dependence of Z″ is also evident in Figure 8, where increasing temperature shifts the peak to higher frequencies. This shift is attributed to the transition from immobile species at lower temperatures to thermally activated vacancies at higher temperatures, facilitating charge transport. Notably, at a higher doping concentration of 5% (Figure 8c), the impedance behavior changes significantly, showing an overall decrease in resistance compared with the 1% and 3% samples. This suggests that at 5% doping, the formation of conductive pathways compensates for the previously dominant charge carrier scattering effects. Similar trends have been reported in ferrite materials, where Co doping initially increases resistance but, beyond a certain concentration, enhances electrical conductivity by promoting electron hopping mechanisms [65].

The observed impedance behavior of Co-doped γ-Fe_2_O_3_ highlights its potential for various electronic applications, where controlled resistivity and relaxation mechanisms are essential. The significant increase in impedance with Co doping, particularly in the 1% and 3% samples, suggests the introduction of localized defect states that act as charge carrier traps, reducing charge mobility. This makes Co-doped γ-Fe_2_O_3_ a promising material for dielectric applications, electrical insulators, and temperature-sensitive resistors. Additionally, the temperature-dependent impedance decrease indicates thermally activated conduction, which can be beneficial in thermistor-based sensors and switching devices. The broadening of Z″ peaks with the increase in doping suggests a more complex relaxation process due to charge carrier scattering, making this material suitable for frequency-dependent electronic components such as impedance-tunable circuits and chemiresistive gas sensors. Interestingly, at 5% doping, a transition from resistive to conductive behavior is observed, indicating the formation of conductive pathways that could enhance electron hopping. This crossover suggests potential applications in varistors, semiconductor components, and mixed ionic–electronic conductors. Furthermore, the tunability of the electrical properties with Co doping makes γ-Fe_2_O_3_ a strong candidate for electromagnetic interference (EMI) shielding and resistive switching memories (RRAM). Overall, the ability to tailor the impedance and relaxation characteristics of γ-Fe_2_O_3_ through controlled Co doping broadens its applicability in advanced electronic and energy storage devices.

Figure 8d provides a comparative analysis of the imaginary impedance (Z″) for pure and Co-doped γ-Fe_2_O_3_ samples with different doping concentrations (1%, 3%, and 5%). The frequency-dependent Z″ behavior exhibits a characteristic relaxation peak for all samples, corresponding to charge carrier polarization and dielectric relaxation. The pure γ-Fe_2_O_3_ sample displays the lowest peak magnitude, confirming its higher electrical conductivity compared with the doped samples. With the increase in Co doping, the relaxation peaks become broader and shift toward higher frequencies, indicating a more complex charge transport mechanism due to enhanced defect states and charge carrier scattering. The 5% Co-doped sample exhibits the highest Z″ values at lower frequencies, suggesting the formation of additional charge trapping centers that impede carrier mobility. However, at higher frequencies, a decrease in Z″ is observed, implying the onset of conduction pathways that facilitate electron hopping. This trend suggests that while moderate Co doping (1% and 3%) primarily increases resistivity by introducing localized states, excessive doping (5%) alters the charge transport dynamics, potentially leading to a crossover toward enhanced conductivity. These findings reinforce the tunability of γ-Fe_2_O_3_’s dielectric properties through controlled Co doping, making it a promising candidate for frequency-dependent electronic applications such as dielectric capacitors, energy storage devices, and impedance-tunable circuits.

##### Nyquist Diagram

**Figure 9:**
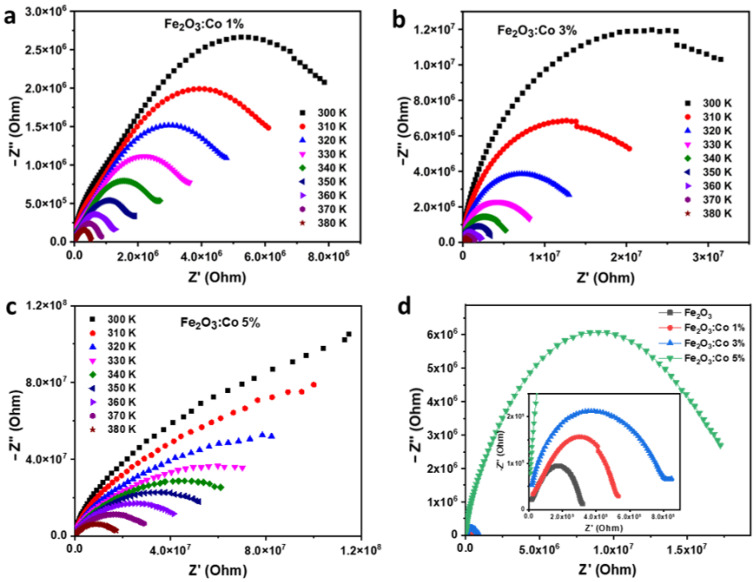
Nyquist diagrams of (**a**) Co1ZO, (**b**) Co3ZO, and (**c**) Co5ZO over a wide temperature range and (**d**) a comparison at 380 K between pure and Co-doped maghemite samples.

The Nyquist and Cole–Cole plots in Figure 9a–c provide essential insights into the impedance contributions from grains and grain boundaries in the Co-doped γ-Fe_2_O_3_ samples [66]. These plots illustrate how both the real (Z′) and imaginary (Z″) components of impedance evolve with the increase in Co doping and temperature. As seen in Figure 9a and Figure 9b, the semicircles corresponding to the Nyquist plots become larger with higher Co content (1% to 3%), indicating an increase in resistivity due to enhanced charge carrier scattering and the introduction of additional localized states. The larger arcs suggest that charge transport is increasingly dominated by grain boundary effects, which typically exhibit higher resistivity than the grain interiors [67].

The addition of Co significantly alters the surface and interface characteristics of the nanoparticles, affecting both charge transport and accumulation layers. Co ions can introduce localized states within the bandgap, trapping charge carriers and reducing their mobility, which explains the observed increase in impedance at lower doping levels (Figure 9a,b) [68]. However, at higher doping concentrations, as seen in Figure 9c, the semicircle diameter decreases, indicating a reduction in resistance. This suggests that beyond a certain Co concentration, the formation of conductive pathways starts to dominate, improving overall charge transport [69]. Such behavior highlights the complex interplay between grains and grain boundaries, demonstrating how doping can either enhance or hinder conductivity depending on concentration and temperature.

These impedance analyses underscore the importance of optimizing Co doping levels for tailoring the electrical properties of γ-Fe_2_O_3_ nanoparticles for specific applications. The findings confirm that Co doping modifies the relaxation mechanisms, resistivity, and overall electrical response, making it a crucial factor in designing materials for sensors, capacitors, and other electronic applications [70].

These findings further emphasize the tunability of Co-doped γ-Fe_2_O_3_ for resistive applications, particularly in semiconductor devices, where controlled impedance is crucial. As depicted in Figure 9d, the comparative Nyquist plot for different doping concentrations (1%, 3%, and 5%) reveals a clear evolution in impedance behavior. The arc diameter increases with Co doping up to 3%, indicating higher resistance due to enhanced charge carrier scattering and the formation of localized defect states. This behavior is attributed to the dominance of grain boundary effects, which impede conductivity at lower doping levels. However, at 5% doping, the semicircle diameter decreases significantly, signaling a reduction in resistance and the onset of conductive pathways. This transition suggests that beyond a critical doping threshold, the increased carrier concentration and percolation effects facilitate charge transport, balancing the previously dominant resistive behavior. The emergence of such conductive pathways underscores the potential of Co-doped γ-Fe_2_O_3_ for applications requiring tunable electrical properties, such as varistors, microelectronic insulating layers, high-temperature sensors, and frequency-dependent electronic components. The ability to tailor impedance through Co doping offers promising prospects for optimizing the material for energy storage devices, resistive switching applications, and electromagnetic interference (EMI) shielding.

## 4. Conclusions

This study explored the synthesis and characterization of pure and cobalt-doped maghemite (γ-Fe_2_O_3_) nanoparticles, highlighting the impact of cobalt incorporation on structural, morphological, and electrical properties. XRD analysis confirmed that cobalt doping preserved the cubic P4_3_32 phase while inducing slight lattice distortions due to ionic substitution, leading to an increase in crystallite size. SEM and TEM imaging further supported these findings, revealing changes in particle shape and size distribution, while EDX confirmed the successful integration of cobalt into the maghemite lattice. Electrical studies demonstrated a distinct correlation between cobalt content and conductivity. DC conductivity analysis indicated semiconducting behavior, with increased Co doping resulting in reduced charge carrier mobility and enhanced resistive properties. The observed increase in activation energy with higher Co concentration suggests a more significant energy barrier for charge transport. Moreover, AC conductivity exhibited a frequency-dependent response, characteristic of small polaron hopping conduction, reinforcing the role of Co in modifying electronic transport mechanisms. Impedance spectroscopy provided further insights into resistive behavior, revealing that both real and imaginary impedance values increased with cobalt doping. This indicates a higher resistive state and reduced charge transport efficiency, which can be beneficial for specific electronic applications. These findings highlight the potential of cobalt-doped maghemite as a tunable material for advanced electronic components, particularly in resistive switching devices and negative temperature coefficient (NTC) thermistors. Additionally, the observed electrical behavior suggests potential applications in energy storage systems, where controlled resistivity and charge transport dynamics are crucial. Overall, this study underscores the significance of fine-tuning cobalt doping concentration to optimize the structural and electrical properties of maghemite nanoparticles, paving the way for their integration into next-generation electronic and energy storage technologies.

## Data Availability

The data supporting the findings of this study are available from the corresponding author upon reasonable request.
